# Anticancer Activity of Ramalin, a Secondary Metabolite from the Antarctic Lichen *Ramalina terebrata*, against Colorectal Cancer Cells

**DOI:** 10.3390/molecules22081361

**Published:** 2017-08-17

**Authors:** Sung-Suk Suh, Tai Kyoung Kim, Jung Eun Kim, Ju-Mi Hong, Trang Thu Thi Nguyen, Se Jong Han, Ui Joung Youn, Joung Han Yim, Il-Chan Kim

**Affiliations:** 1 Division of Polar Life Sciences, Korea Polar Research Institute, Incheon 21990, Korea; sung-suk.suh@kopri.re.kr (S.-S.S); tkkim@kopri.re.kr (T.K.K.); je2202@kopri.re.kr (J.E.K.); wnal5555@kopri.re.kr (J.-M.H.); hansj@kopri.re.kr (S.J.H.); ujyoun@kopri.re.kr (U.J.Y.); jhyim@kopri.re.kr (J.H.Y.); 2 Department of Polar Life Science, University of Science and Technology, Incheon 21990, Korea; 3 Department of Pharmacy, Graduate School, Sungkyunkwan University, Suwon 16419, Korea; 4 Department of Pharmacological Medical and Agronomical Biotechnology, University of Science and Technology of Hanoi, Hanoi 100000, Vietnam; buffson192@gmail.com

**Keywords:** colorectal cancer, ramalin, Antarctic lichen, cell cycle arrest, HCT116

## Abstract

Colorectal cancer is a leading cause of death worldwide and occurs through the highly complex coordination of multiple cellular pathways, resulting in carcinogenesis. Recent studies have increasingly revealed that constituents of lichen extracts exhibit potent pharmaceutical activities, including anticancer activity against various cancer cells, making them promising candidates for new anticancer therapeutic drugs. The main objective of this study was to evaluate the anticancer capacities of ramalin, a secondary metabolite from the Antarctic lichen *Ramalina terebrata*, in the human colorectal cancer cell line HCT116. In this study, ramalin displayed concentration-dependent anticancer activity against HCT116 cells, significantly suppressing proliferation and inducing apoptosis. Furthermore, ramalin induced cell cycle arrest in the gap 2/mitosis (G2/M) phase through the modulation of hallmark genes involved in the G2/M phase transition, such as tumour protein p53 (*TP53*)*,* cyclin-dependent kinase inhibitor 1A (*CDKN1A*)*,* cyclin-dependent kinase 1 (*CDK1*) and cyclin B1 (*CCNB1*). At both the transcriptional and translational level, ramalin caused a gradual increase in the expression of *TP53* and its downstream gene *CDKN1A*, while decreasing the expression of *CDK1* and *CCNB1* in a concentration-dependent manner. In addition, ramalin significantly inhibited the migration and invasion of colorectal cancer cells in a concentration-dependent manner. Taken together, these data suggest that ramalin may be a therapeutic candidate for the targeted therapy of colorectal cancer.

## 1. Introduction

Colon cancer is one of the leading causes of morbidity and mortality, and is the third most common cancer in both men and women worldwide. According to an annual report by the American Cancer Society, in 2012, approximately 1.2 million new cases of colorectal cancer were diagnosed, and colorectal cancer resulted in more than 774,000 deaths. One of the hallmarks of cancer is that abnormal cancer cells can penetrate into adjacent parts of the body and spread to other organs, in a process called metastasis [1]. In terms of patient survival, metastatic colorectal cancer has a very poor prognosis compared to other cancers. In fact, approximately 20–25% of patients with colorectal cancer have progressed to metastasis at the time of diagnosis, and approximately 50% of all patients with colorectal cancer eventually develop metastatic cancers [2]. Thus, an urgent need exists for agents that can treat colon cancer and prevent its metastasis.

Recently, many studies on the causes of colorectal cancer have shown that carcinogenesis occurs through a complex pathogenic process via three different pathways, namely, chromosomal instability, microsatellite instability (MSI) and 5′-C-phosphate-G-3′ (CpG) island methylation [3,4]. Chromosomal instability plays a fundamental part in the development of sporadic colorectal tumorigenesis through the deregulation of tumorigenesis-related genes, tumor suppressors and oncogenes such as adenomatous polyposis coli (*APC*), kirsten rat sarcoma 2 viral oncogene homolog (*KRAS*) and tumour protein p53 (*TP53*) [5]. Colorectal cancer begins with mutation in the *APC* tumor suppressor gene, followed by the mutational activation and inactivation of the oncogene *KRAS* and the tumor suppressor gene *TP53*, respectively. Usually, these gene mutations tend to cause numerous colon polyps, which can be categorized as adenomas, hyperplastic polyps and inflammatory polyps, and can potentially progress to colon cancer [6]. MSI is caused by impaired DNA mismatch repair that causes inactivation mutations in DNA mismatch repair genes such as *hMSH2*, *hMSH6*, *MSH3* and *MLH1*, leading to tumorigenesis in the colon [7]. For example, hypermethylation of the *MLH1* promoter is found in over 80% of MSI-mediated sporadic colorectal cancers [4,7]. In the CpG island methylation pathway, the promoter sequences of carcinogenesis-related genes are significantly methylated, resulting in the transcriptional inactivation of tumor suppressor genes such as *CDKN2A* and *TIMP3* [8,9]. Recently, other pathways have been implicated in the tumorigenesis of colon cancer, including inflammation and microRNA (miRNA) pathways [10].

The lack of knowledge regarding the mechanisms underlying colorectal cancer development and progression has hampered the development of targeted anticancer agents. However, despite this limitation, a number of clinical trials investigating chemotherapeutic agents have been conducted in patients with colorectal cancers, and currently, 24 drugs approved by the Food and Drug Administration (FDA) are undergoing clinical trials for colorectal cancer therapy. Furthermore, combination therapies with eight anticancer agents, such as irinotecan or oxaliplatin in combination with leucovorin, significantly improve the overall survival of patients with colorectal cancers. In addition, many scientists have recently investigated the effects of particular exposures such as environmental, genetic factors and chemotherapeutic agents on carcinogenesis by examining the molecular pathologic changes of tumor initiation or progression. These exposures can be prognostic and predictive biomarkers in colorectal cancer. This new field of scientific research represents molecular pathologic epidemiology (MPE), which can provide new insights to better understand how specific exposures affect the carcinogenic process in colorectal cancer, and help to develop personalized therapies to target specific molecules or pathways [11,12].

**Figure 1:**
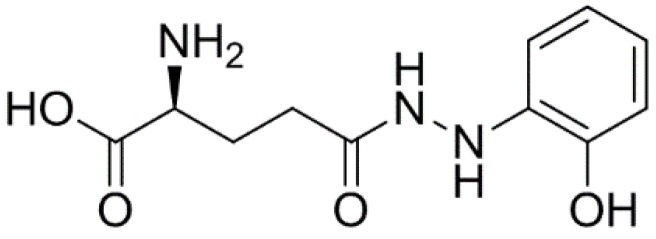
The chemical structure of ramalin.

In recent years, there has been an increasing interest in lichens as a potential source of pharmacologically bioactive agents for therapeutic treatments [13,14,15]. Lichens are symbiotic organisms consisting of a fungus and algae or cyanobacteria, and produce a variety of bioactive metabolites, including a large number of phenolic compounds such as depsides, depsidones and dibenzofurans. More than 1000 metabolites have been identified as lichen-derived compounds, and these compounds have a wide range of biological activities such as anti-inflammatory, antioxidant, cytotoxic, and antiproliferative activities [16]. Recent studies have shown that ramalin, a secondary metabolite derived from the Antarctic lichen *Ramalina terebrata* (Figure 1), has significant antioxidant and anti-inflammatory activities [17,18]. However, the biological activity of ramalin in carcinogenesis and cancer therapy has not been well investigated. Therefore, in this study, we investigated the anticancer activities of ramalin in the human colorectal cancer cell line HCT116. Our results showed that ramalin inhibits proliferation, invasion and migration in colon cancer cells, and induces cell cycle arrest and apoptosis. These data suggest that ramalin may be a promising candidate as an anticancer drug in the targeted therapy of colorectal cancer.

## 2. Results and Discussion

### 2.1. Antiproliferative Activity of Ramalin

**Figure 2:**
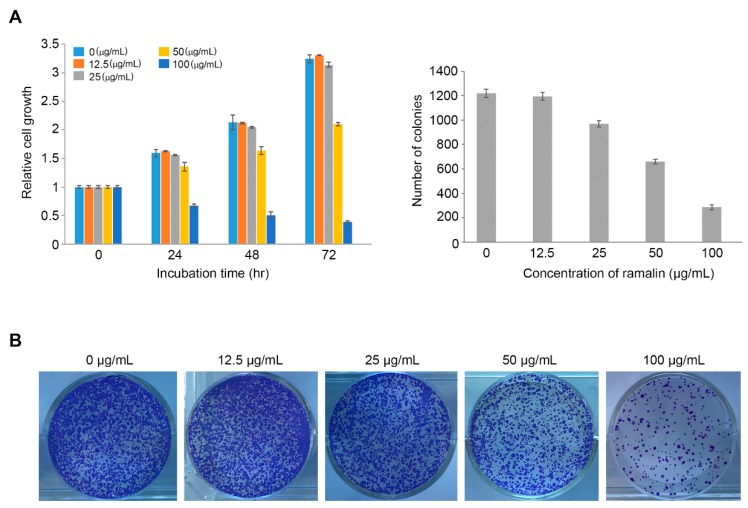
Effect of ramalin on cell proliferation in human colorectal cancer cells (HCT116). Ramalin significantly suppressed proliferation of HCT116 cells in a time-dependent manner at the highest concentrations used (50 and 100 μg/mL) (**A**) relative to the untreated control cells. Ramalin treatment significantly decreased the number of colonies in a concentration-dependent manner (**B**) relative to the untreated control cells.

To determine if ramalin exerted a cytotoxic effect on colorectal cancer cells, we performed a cell proliferation assay in HCT116 cells, with different concentrations of ramalin (0, 12.5, 25, 50 and 100 μg/mL) and different incubation times (0, 24, 48 and 72 h). The proliferation of HCT116 cells was gradually but significantly inhibited by 50 and 100 μg/mL—but not 12.5 and 25 μg/mL—of ramalin in a time-dependent manner, with the highest concentration (100 μg/mL) showing a stronger inhibitory effect than the lower 50 μg/mL concentration (Figure 2A). Next, to investigate the effect of ramalin on the colony-forming capacity of colon cancer cells, HCT116 cells were seeded at 1000 cells/mL in 6-well plates containing different concentrations of ramalin (0–100 μg/mL). As shown in Figure 2B, ramalin treatment significantly decreased the number of colonies in a concentration-dependent manner at all concentrations except the low 12.5 μg/mL concentration, suggesting that ramalin has a strong inhibitory effect on colony formation in HCT116 cells. This is consistent with the findings of a previous study showing that ramalin exerted a cytotoxic effect against human breast cancer cells at high concentrations (50 and 100 μg/mL) [19], causing a concentration-dependent decrease in cell proliferation. However, in breast cancer cells, ramalin could suppress cell proliferation even at low concentrations, which is in contrast to the lack of effect on colorectal cancer cells at lower concentrations observed in the present study. These data suggest that ramalin may possess different inhibitory capacities against different types of cancers.

### 2.2. Ramalin-Induced Cell Cycle Arrest through the Modulation of Cell Cycle Marker Genes

**Figure 3:**
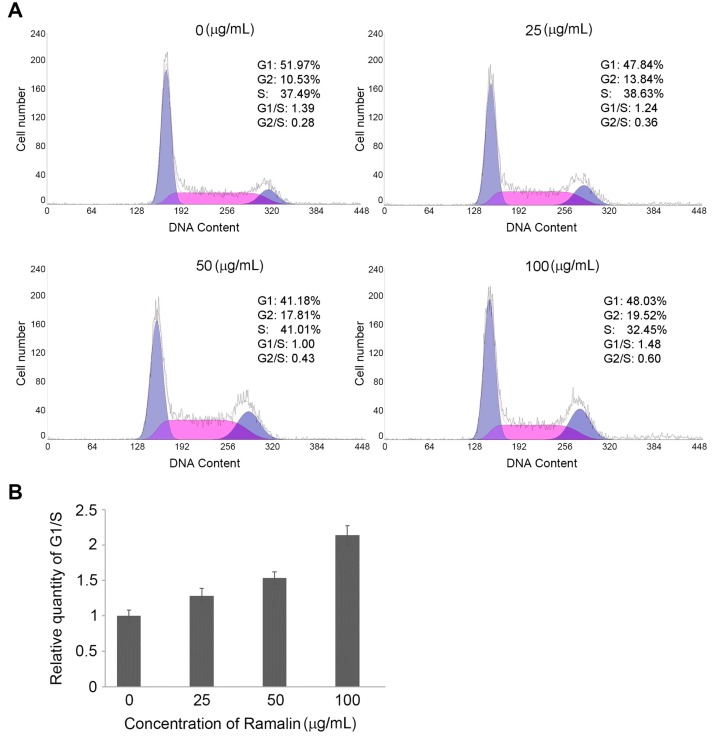
Effect of ramalin on the cell cycle in HCT116 cells. Ramalin induced cell cycle arrest in the gap 2/mitosis (G2/M) phase in a concentration-dependent manner (**A**,**B**), relative to the untreated control cells.

**Figure 4:**
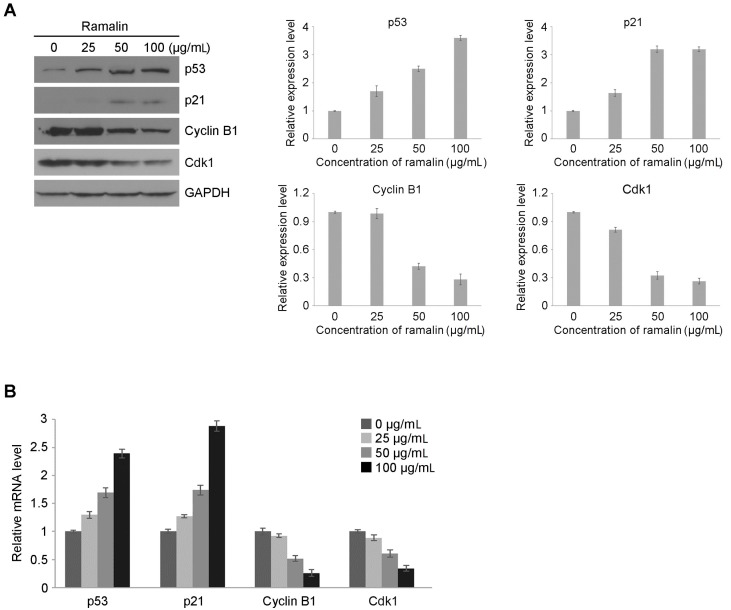
Expression of genes involved in the G2/M transition in HCT116 cells. At both translational (**A**) and transcriptional (**B**) levels, ramalin significantly increased the expression of *TP53* and *p21* in a concentration-dependent manner, whereas *cyclin B1* and *CDK1* expression was significantly decreased.

Cell cycle signaling is responsible for controlling cell proliferation through tightly controlled regulatory systems via a range of growth factor signaling pathways such as the mitogen-activated protein kinase and wingless/integrated (Wnt)-signaling pathways [20,21]. These pathways are controlled by the sequential activation of a number of protein kinases, known as cyclin-dependent kinases (CDKs), through the formation of complexes with various cyclins [22]. To verify whether ramalin inhibited cell proliferation by inducing cell cycle arrest, we performed flow cytometry analysis in HCT116 cells treated with different concentrations of ramalin (0–100 μg/mL). As shown in Figure 3A, a significant concentration-dependent increase in the percentage of cells in the gap 2/mitosis (G2/M) phase was observed along with a decrease in the population of cells in the synthesis (S) phase of the cell cycle. In the control cells, 51.97% were in the gap 0/gap 1 (G0/G1) phase, 37.49% were in the S phase and 10.53% were in the G2/M phase. However, the percentage of cells in the G1 phase gradually decreased with increasing concentrations of ramalin up to 50 μg/mL (51.97% at 0 μg/mL, 47.84% at 25 μg/mL and 41.18% at 50 μg/mL), but rebounded at 100 μg/mL (48.03%). The percentage of cells in the S phase was increased at the same concentrations (37.49% at 0 μg/mL, 38.63% at 25 μg/mL and 41.01% at 50 μg/mL), whereas the percentage of cells in the G2/M phase was increased up to the highest concentration of 100 μg/mL (Figure 3B). These data provided strong evidence of cell cycle arrest at the G2/M phase, and thus, of ramalin-induced inhibition of cell division in colon cancer cells. The cell cycle is composed of multiple phases including the S, G2, M and G1 phases, which are tightly regulated by sequential biochemical events such as the phosphorylation and degradation of heterodimeric protein kinases called cyclins [22]. For example, the cyclin B1-dependent activation of cyclin-dependent kinase 1 (CDK1) is responsible for the G2/M transition of the cell cycle. In addition, the tumour protein (*TP53*) tumor suppressor and its downstream gene *p21* play a crucial role in the regulation of multiple cell cycle checkpoints, including the G2/M transition [23]. Notably, *TP53*-dependent G2/M arrest occurs through a significant decrease in the levels of cyclin B1 and CDK1. Using flow cytometry, we observed that ramalin induced G2/M phase arrest in HCT116 cells. Therefore, to examine the mechanism underlying ramalin-mediated cell cycle arrest, we investigated the effect of ramalin on the expression patterns of genes involved in the G2/M transition, including *TP53*, *p21*, *CDK1* and *cyclin B1* (*CCNB1*). Immunoblot assays (Figure 4A) revealed that ramalin treatment significantly increased the protein levels of *TP53* and p21 in a concentration-dependent manner, whereas protein expression of CCNB1 and CDK1 was decreased. Consistently, the levels of *TP53* and p21 messenger RNAs (mRNAs) were higher in ramalin-treated cells, whereas *CCNB1* and *CDK1* expression was considerably downregulated (Figure 4B). These data suggest that the ramalin-induced inhibition of cell proliferation is mediated, at least in part, through the induction of G2/M phase arrest via regulation of the expression of marker genes that are necessary for the G2/M transition.

### 2.3. Ramalin-Mediated Induction of Apoptosis in HCT116 Cells

**Figure 5:**
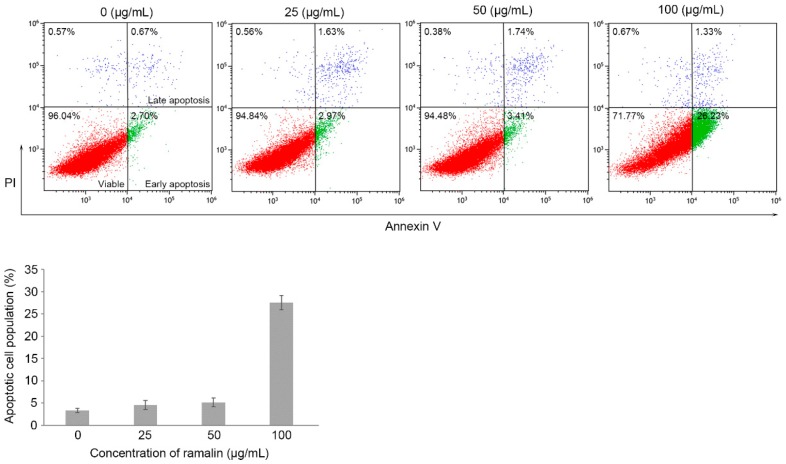
Ramalin-mediated apoptosis in HCT116 cells. Ramalin induced apoptosis at the highest concentration used (100 μg/mL) significantly increased the proportion of apoptotic cells relative to that in the untreated control cells.

Cancer cells undergo uncontrolled cell division without programmed cell death or apoptosis [24]. Therefore, despite their many therapeutic limitations, cancer treatments have focused on apoptosis as one of their main targets [25,26]. Lipid composition changes in the plasma membrane are one of the characteristic features of the apoptotic process. For example, in cells undergoing apoptosis, phosphatidylserine, normally located on the inner surface of the plasma membrane, flips rapidly across to the outer surface [27], where it can be detected by flow cytometry through binding to the annexin V protein. In the present study, we hypothesized that a significant proportion of the antiproliferation effect of ramalin in HCT116 cells was due to apoptosis. To test this hypothesis, we performed flow cytometric analysis to determine apoptotic cell populations in ramalin-treated HCT116 cells, using annexin V-binding to identify cells undergoing early and late apoptosis. As shown in Figure 5, the population of HCT116 cells undergoing apoptosis increased significantly at the highest concentration of ramalin (27.56% at 100 μg/mL), whereas the population of apoptotic cells at low ramalin concentrations was low and not significantly higher than that of the untreated group. Many studies have revealed that the early apoptotic pathway is tightly controlled by multiple proteins such as *TP53*, B-cell lymphoma 2 (Bcl-2), and Bcl-2-associated x (Bax) [25,28]. In particular, the *TP53* tumor suppressor gene plays a crucial role in regulating the response to numerous extrinsic or intrinsic apoptotic signals, and stimulates apoptosis for the maintenance of cell integrity. Therefore, many human tumors harboring *TP53* mutations are capable of evading apoptosis, and exhibit distinct drug resistance compared with human cancers with functional *TP53*. Furthermore, *TP53* activation in HCT116 cells can induce cell cycle arrest and apoptosis in response to genotoxic stress [29]. In the present study, we observed that ramalin treatment resulted in a significant increase in *TP53* expression in HCT116 cells (Figure 4), suggesting that the ramalin-induced apoptosis may occur, at least in part, via a *TP53*-dependent apoptotic pathway. Consistent with this, a previous study revealed that Antarctic lichen-derived ramalin induced apoptosis via a *TP53*-dependent pathway in breast cancer cells with wild-type *TP53* [19]. Interestingly, ramalin-mediated apoptosis mainly occurs in the early apoptotic pathway in both breast and colon cancer cells. Taken together, these data suggest that ramalin may be involved in the early signal transduction of apoptotic pathways by activating *TP53*.

### 2.4. Ramalin-Mediated Inhibition of Cellular Migration and Invasion 

**Figure 6:**
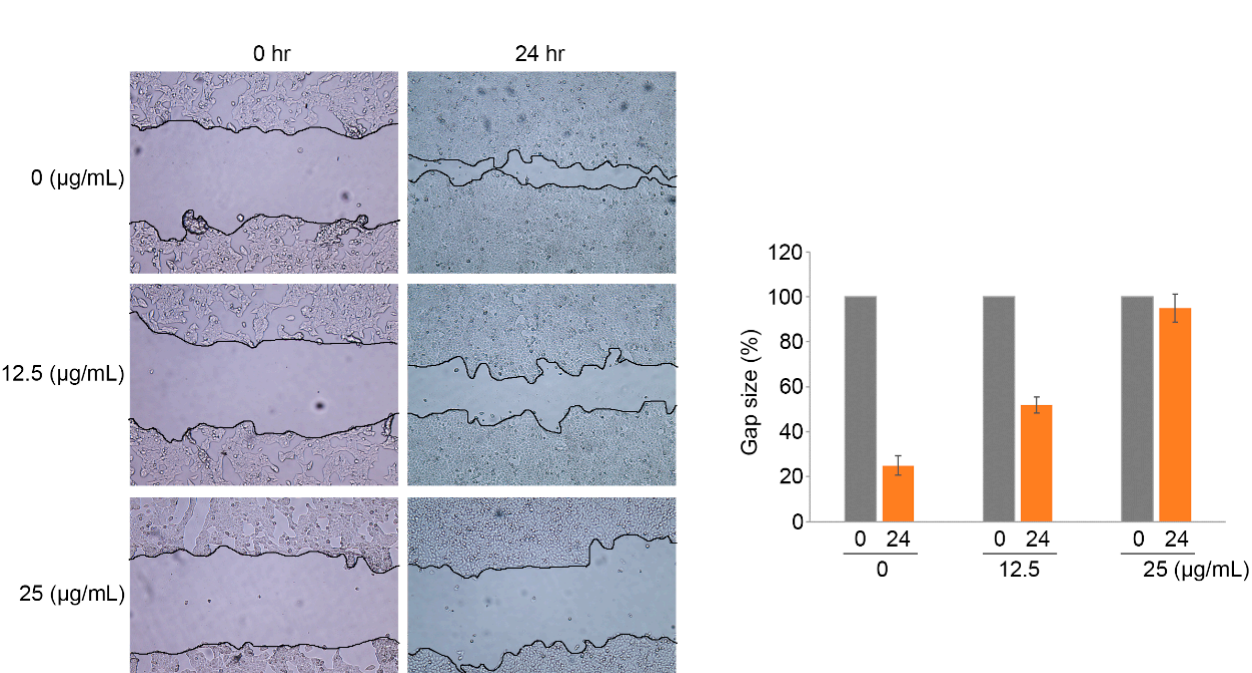
Effect of ramalin on wound healing in HCT116 cells. Ramalin significantly suppressed the wound-healing capacity of HCT116 cells in a concentration-dependent manner, relative to the untreated control cells.

**Figure 7:**
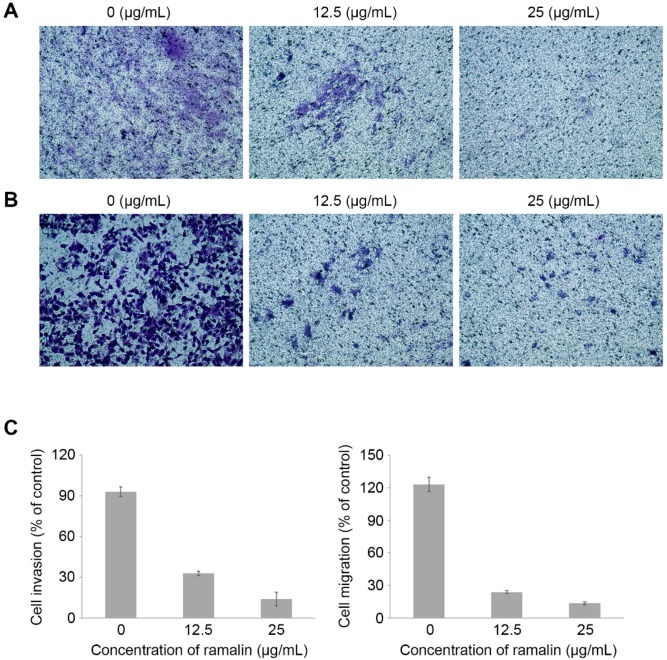
Anti-invasive and migratory activity of ramalin in HCT116 cells. Ramalin significantly suppressed invasion (**A**) and migration (**B**) of HCT116 colorectal cancer cells in a concentration-dependent manner (**C**), relative to the untreated control cells.

Although previous studies have revealed many biological effects of ramalin, such as antioxidant activity and anticancer activity [17,18,19], the wound-healing effects of ramalin on cancer cells have not yet been reported. The wound-healing effects of ramalin at the concentrations that induced no cytotoxicity (12.5 and 25 μg/mL) were evaluated in HCT116 cells by using a 3-(4,5-dimethylthiazol-2-yl)-2,5-diphenyltetrazoliumbromide (MTT) cell viability assay (Figure 2A). Wound healing is a complex procedure that includes inflammatory, proliferative and remodeling phases. Migration of cancer cells is essential for wound healing and angiogenesis. To investigate the role of ramalin in the regulation of wound healing, we treated HCT116 cells with non-cytotoxic doses of ramalin, and examined its inhibitory effect on the wound-healing process. As shown in Figure 6, ramalin significantly inhibited wound healing in HCT116 cells in a concentration-dependent manner. In particular, the migration of cancer cells was almost completely inhibited by the highest concentration of ramalin (25 μg/mL), which resulted in migration similar to that of cells in the control group. These results indicated that ramalin suppresses the wound-healing process in human colorectal cancer. Cancer metastasis occurs via multiple sequential processes involving intravasation, circulation in the blood, extravasation and cell growth at a secondary site; migration and invasion of cancer cells are an essential part of these processes [1]. Recently, many scientists have demonstrated that metastasis can be inhibited by suppressing the migratory and invasive capacity of cancer cells [1]. Therefore, we used the Boyden chamber system to investigate the inhibitory effects of ramalin on cancer cell migration and invasion. As shown in Figure 7, both migration and invasion of HCT116 cells were significantly suppressed by ramalin in a concentration-dependent manner. This is the first report to provide evidence suggesting that ramalin can effectively regulate cancer metastasis by dramatically suppressing migration and invasion.

## 3. Materials and Methods

### 3.1. Cell Culture and Cell Growth Inhibition Assay

HCT116 cells used in this study were obtained from the American Type Culture Collection (ATCC, Manassas, VA, USA), and grown in Dulbecco’s modified Eagle’s medium containing 10% fetal bovine serum and 1% penicillin–streptomycin. Cells were cultured in a 5% CO_2_ humidified incubator at 37 °C, seeded in a 96-well plate in triplicate at a density of 5 × 10^3^ cells/well, and incubated at 37 °C in a humidified CO_2_ incubator. After overnight incubation, the cells were treated with different concentrations (12.5, 25, 50, and 100 μg/mL) of ramalin at 37 °C in a humidified CO_2_ incubator. The ramalin concentration range was determined based on the results of the cytotoxicity assay. Cell proliferation was measured for 72 h. At every 24 h interval, 20 μL MTS (3-(4,5-dimethylthiazol-2-yl)-5-(3-carboxymethoxyphenyl)-2-(4-sulfophenyl)-2*H*-tetrazolium) (Promega, Madison, WI, USA) was added into each well. After 1 h of incubation, the optical density was measured using a Multilabel Counter (Bio-Rad Laboratories, Hercules, CA, USA). In addition, changes in cell morphology were observed under a Nikon D700 phase–contrast inverse microscope (Nikon, Melville, NY, USA).

### 3.2. Cell Cycle Analysis

HCT116 cells were treated with different concentrations (0, 25, 50, 100 μg/mL) of ramalin and then incubated for 24 h. Cells were fixed in 70% ice-cold ethanol and stored at 4 °C for at least 12 h. Next, the cells were washed in phosphate-buffered saline (PBS), rehydrated and resuspended in a solution of PBS containing 50 g/mL propidium iodide and 50 g/mL RNase A. Cellular DNA content was determined by flow cytometry (Becton Dickinson, Holdrege, NE, USA).

### 3.3. RNA Extraction and Reverse Transcription Polymerase Chain Reaction *(*RT-PCR*)*

Total RNA was extracted using TRIzol Reagent (Invitrogen, Waltham, MA, USA) following the manufacturer’s instruction. Specifically, the pellet obtained from 5 × 10^6^ cells was lysed with 1 mL TRIzol solution. At the end of the extraction, the isolated RNA was dissolved in 35 μL of RNase-free water and incubated for 10 min at 55 °C. An aliquot of 5 μg RNA was then used for complementary DNA (cDNA) synthesis using the Super Script first strand cDNA synthesis kit (Invitrogen, Waltham, MA, USA), and the samples were analyzed using SYBR green real-time PCR master mixes (ThermoFisher Inc., Waltham, MA, USA) with gene expression primers. The primers used in this study were as follows: p53, 5′-CCGCAGTCAGATCCTAGCG-5′ and 5′-AATCATCCATTGCTTGGGACG-5′; p21, 5′-TGCCGAAGTCAGTTCCTTGT-3′ and 5′-CATGGGTTCTGACGGACATC-3′; Cyclin B1, 5′-AAAGGCGTAACTCGAATGGA-3′ and 5′-CCGACCTTTTATTGAAGAGCA-3′; Cdk1, 5′-CCGAAATCTGCCAGTTTGAT-3′ and 5′-CTGGCCAGTTCATGGATTCT-3′; GAPDH, 5′-GAAGGTGAAGGTCGGAGTC-3′ and 5′-GAAGATGGTGATGGATTTC-3′.

### 3.4. Western Blot Analysis

Cells were lysed on ice in a radioimmune precipitation assay (RIPA) buffer (Sigma, St. Louis, MO, USA) and constantly agitated for 30 min. The cell lysate was centrifuged in a microcentrifuge at 4 °C, and the supernatant was aspirated and placed in a fresh tube on ice. Equal amounts of total protein (30 μg) were loaded into wells of a 4–20% precast gel (Bio-Rad Laboratories), and electrophoresed for 1 h at 100 V. The separated proteins were transferred from the gel to a nitrocellulose membrane. The membrane was blocked for 1 h at room temperature using 5% milk solution, incubated overnight at 4 °C, and then analyzed with primary antibodies against *TP53*, p21, CDK1 and cyclin B1 (Cell Signaling, Beverly, MA, USA). After probing the membrane with horseradish peroxidase-conjugated secondary antibody IgG (Santa Cruz Biotechnology, Dallas, TX, USA) for 1 h at room temperature, the membrane was developed using a chemiluminescence kit (Amersham Pharmacia, Pittsburgh, PA, USA).

### 3.5. Apoptosis Assay

A flow-cytometric assay system (BD Biosciences, San Jose, CA, USA) was used to measure the binding of annexin V–fluorescein isothiocyanate (FITC) to phosphatidylserine residues on the outer membrane of apoptotic cells. Ramalin-treated cells were washed twice with cold PBS and resuspended in 1× binding buffer at a concentration of ~1 × 10^6^ cells/mL. Next, 100 μL of the solution (~1 × 10^5^ cells/mL) was transferred to a 5 mL culture tube, and annexin V-FITC (5 μL) and propidium iodide (2 μL) solutions were added to each tube, followed by gentle mixing with a pipette. After incubation at room temperature for 20 min, a flow cytometry assay was performed to evaluate changes in the proportion of apoptotic cells in response to ramalin.

### 3.6. Wound-Healing Assay

HCT116 cells were seeded in 6-well plates at a density of 0.5 × 10^6^ cells/mL and cultured for 24 h at 37 °C in a humidified 5% CO_2_ incubator. A linear scratch was made in the confluent monolayer in each well by gently scraping the surface with a sterile p200 pipette tip. Cells were washed two times with PBS to remove cellular debris cells, and then fresh media containing ramalin was added into each well. After 24 h, images of migrated cells in the wound area were captured using a digital camera. All scratch assays were performed in triplicate.

### 3.7. Colony-Forming Assay

HCT116 cells were seeded in a 6-well plate at a density of 1 × 10^3^ cells/well. After 24 h incubation for cell adherence, the cells were exposed to different concentrations of ramalin (12.5, 25, 50, and 100 µg/mL) for 12 h. The ramalin-treated cells were then incubated in a regular medium until colonies were viable. Cells were fixed, stained and counted as described in a previous study [30].

### 3.8. Invasion and Migration Assays

Migration and invasion assays were performed using a 24-well migration and invasion plate containing a polycarbonate membrane insert (Cell Biolabs, San Diego, CA, USA). Media containing 10% fetal bovine serum (FBS) (500 μL) was added to the bottom chamber of the invasion or migration plate. Cells were suspended at a concentration of 1.0 × 10^6^ cells/mL in serum-free media. The cell suspension (300 μL) was added to the top chamber, treated directly with different concentrations of ramalin (12.5 and 25 μg/mL) and maintained in a cell culture incubator for 48 h. The cells were stained using a Cell Stain Solution (400 μL) and photographed.

### 3.9. Statistical Analysis

The results are presented as mean ± standard deviation values for three independent biological experiments. Statistical significant differences between each treated group and the control group were determined by one-way analysis of variance followed by Student’s *t*-test; *p*-values < 0.05 were considered statistically significant.

## 4. Conclusions

In the present study, we showed that ramalin, a secondary metabolite derived from the Antarctic lichen *Ramalina terebrata*, has significant anticancer activities in the colon cancer cell line HCT116. These activities included antiproliferation, induction of cell cycle arrest and apoptosis, and inhibition of migration and invasion. To our knowledge, this is the first study to show that ramalin-mediated antiproliferative activity in colon cancer cells occurs—at least in part—by inducing cell cycle arrest, and that ramalin may impede colon cancer metastasis via significant inhibition of migration and invasion. Further studies are required to evaluate the therapeutic effects of ramalin on other cancer cell lines or with in vivo xenograft models for application in clinical trials, and to investigate the effect of ramalin on the molecular events and multiple pathogenic processes, including chromosomal instability, that are involved in colorectal carcinogenesis. Taken together, these comprehensive data indicate that ramalin plays an important role in modulating colon tumorigenesis, and provide novel insight to better understand its role as an anticancer agent.
